# A large-scale survey of the novel 15q24 microdeletion syndrome in autism spectrum disorders identifies an atypical deletion that narrows the critical region

**DOI:** 10.1186/2040-2392-1-5

**Published:** 2010-03-19

**Authors:** L Alison McInnes, Alisa Nakamine, Marion Pilorge, Tracy Brandt, Patricia Jiménez González, Marietha Fallas, Elina R Manghi, Lisa Edelmann, Joseph Glessner, Hakon Hakonarson, Catalina Betancur, Joseph D Buxbaum

**Affiliations:** 1 Seaver Autism Center for Research and Treatment, Department of Psychiatry, Mount Sinai School of Medicine, New York, NY 10029, USA; 2 Department of Genetics and Genomic Sciences, Mount Sinai School of Medicine, New York, NY 10029, USA; 3 Laboratory of Molecular Neuropsychiatry, Mount Sinai School of Medicine, New York, 10029 NY, USA; 4 INSERM U952, 75005 Paris, France; 5 CNRS UMR 7224, 75005 Paris, France; 6 UPMC Univ Paris 06, 75005 Paris, France; 7 Hospital Nacional de Niños Dr Sáenz Herrera, CCSS, Child Developmental and Behavioral Unit, San José, Costa Rica; 8 Department of Disability and Human Development, University of Illinois at Chicago, Chicago, IL 60608, USA; 9 Center for Applied Genomics, Children's Hospital of Philadelphia, Philadelphia, PA 19104, USA; 10 Department of Neuroscience, Mount Sinai School of Medicine, New York, NY 10029, USA; 11 The Charles R. Bronfman Institute for Personalized Medicine, Mount Sinai School of Medicine, New York, 10029 NY, USA

## Abstract

**Background:**

The 15q24 microdeletion syndrome has been recently described as a recurrent, submicroscopic genomic imbalance found in individuals with intellectual disability, typical facial appearance, hypotonia, and digital and genital abnormalities. Gene dosage abnormalities, including copy number variations (CNVs), have been identified in a significant fraction of individuals with autism spectrum disorders (ASDs). In this study we surveyed two ASD cohorts for 15q24 abnormalities to assess the frequency of genomic imbalances in this interval.

**Methods:**

We screened 173 unrelated subjects with ASD from the Central Valley of Costa Rica and 1336 subjects with ASD from 785 independent families registered with the Autism Genetic Resource Exchange (AGRE) for CNVs across 15q24 using oligonucleotide arrays. Rearrangements were confirmed by array comparative genomic hybridization and quantitative PCR.

**Results:**

Among the patients from Costa Rica, an atypical *de novo *deletion of 3.06 Mb in 15q23-q24.1 was detected in a boy with autism sharing many features with the other 13 subjects with the 15q24 microdeletion syndrome described to date. He exhibited intellectual disability, constant smiling, characteristic facial features (high anterior hairline, broad medial eyebrows, epicanthal folds, hypertelorism, full lower lip and protuberant, posteriorly rotated ears), single palmar crease, toe syndactyly and congenital nystagmus. The deletion breakpoints are atypical and lie outside previously characterized low copy repeats (69,838-72,897 Mb). Genotyping data revealed that the deletion had occurred in the paternal chromosome. Among the AGRE families, no large 15q24 deletions were observed.

**Conclusions:**

From the current and previous studies, deletions in the 15q24 region represent rare causes of ASDs with an estimated frequency of 0.1 to 0.2% in individuals ascertained for ASDs, although the proportion might be higher in sporadic cases. These rates compare with a frequency of about 0.3% in patients ascertained for unexplained intellectual disability and congenital anomalies. This atypical deletion reduces the minimal interval for the syndrome from 1.75 Mb to 766 kb, implicating a reduced number of genes (15 versus 38). Sequencing of genes in the 15q24 interval in large ASD and intellectual disability samples may identify mutations of etiologic importance in the development of these disorders.

## Background

The widespread use of genomic DNA array-based technologies to detect whole genome copy number variations (CNVs) has led to the identification of novel recurrent genomic disorders associated with syndromic or nonsyndromic intellectual disability [1]. The 15q24 microdeletion syndrome was first described by Sharp *et al. *in 2007 in four subjects sharing clinical characteristics and overlapping *de novo *deletions [2]. Phenotypic features include mild to moderate developmental delay, characteristic facial features (high anterior hairline, broad medial eyebrows, hypertelorism, downslanting palpebral fissures, broad nasal base, long smooth philtrum, and full lower lip), growth retardation, hypotonia, joint laxity, digital abnormalities and genital abnormalities [2]. The recurrent deletions, ranging from 1.7 to 3.9 Mb, result from non-allelic homologous recombination mediated by low-copy repeat (LCR, also called segmental duplication) clusters in 15q24, termed BP1, BP2 and BP3 [2]. Sharp *et al. *defined a minimal critical region between BP1 and BP2 that spanned 1.75 Mb of genomic sequence. More recently, El-Hattab *et al. *[3] reported four additional deletion cases, which implicated the same 1.75 Mb minimal critical region in the syndrome and described two other LCR clusters that mediate the formation of alternative-sized rearrangements, termed LCR 15q24A, 15q24B (BP1), 15q24C, 15q24D (BP2) and 15q24E (BP3). Three other subjects with 15q24 deletion have been reported recently, further expanding the phenotypic spectrum [4-6]. In addition, there have been four reported 15q24 duplications that involve the same LCRs and share some clinical features with cases of 15q24 microdeletion [3,7].

Genetic variants that present with developmental delay can often be associated with an autism spectrum disorder (ASD). Smith *et al. *described a girl with autism, developmental delay and mild dysmorphism carrying what was first identified as an interstitial deletion of chromosome 15q22-q23 according to fluorescent *in situ *hybridization (FISH) analysis [8], but subsequent microarray mapping revealed to be a 15q24 deletion overlapping the genomic region identified by Sharp *et al. *[2] (Moyra Smith, personal communication). Furthermore, a recent analysis of 427 unrelated subjects with ASD reported a 15q24 *de novo *deletion in a boy with ASD, intellectual disability, severe dysmorphism, scoliosis and diaphragmatic hernia [9]. The deletion was validated by subsequent quantitative (q)PCR and karyotyping, and found to encompass 4.29 Mb.

In this study, we surveyed two ASD cohorts comprising subjects from 958 unrelated families to estimate the frequency of 15q24 deletions in ASD, and mapped 15q24 deletions in these cohorts as a means of generating a plausible list of genes that may contribute to the phenotypes associated with the 15q24 deletion syndrome.

## Methods

The study was approved under the guidelines of the Ministry of Health of Costa Rica, the ethics committee of the National Children's Hospital in San José (Hospital Nacional de Niños, HNN) and the institutional review board at Mount Sinai School of Medicine in accordance with the Declaration of Helsinki.

### Subjects

Subjects were derived from two datasets: a genetic study of autism in an isolated founder population in the Central Valley of Costa Rica (CVCR) (n = 173) and the Autism Genetic Resource Exchange (AGRE) cohort (n = 1336 subjects with ASD from 785 families). The CVCR study, initiated by one of the authors (LAM), including recruitment and assessment, has been described in detail previously [10,11]. This sample comprised 153 males and 20 females and all were sporadic cases (that is, only one affected child in the family). Families of individuals with a known or possible diagnosis of ASD contacted the HNN or were contacted by the Costa Rican research team (PJG, head). If they expressed interest in the study, they were formally asked to participate using established informed consent criteria. All interviews and examinations took place at the Neurodevelopmental Unit of the HNN, where parents were interviewed by an experienced pediatrician (PJG) using the Autism Diagnostic Interview-Revised (ADI-R) [12], and the Autism Diagnostic Observation Schedule (ADOS) [13] was administered. Both assessments were videotaped for independent scoring by the best estimator (ERM). IQ tests appropriate for the age and level of verbal communication of the subjects were administered, as were the Vineland Adaptive Behavioral Scales [14]. A complete medical and neurological examination was performed, including a dermatological examination under Wood's lamp. Subjects were assessed for dysmorphic features and a full panel of photographs was taken for further evaluation by a clinical geneticist. Blood samples were taken from subjects and parents for DNA extraction and transformation into cell lines. The parents of patient AU008 reported here provided written informed consent for publication of clinical data, including photographs.

The AGRE cohort is a well-characterized sample that has been used in numerous studies. Recently, the AGRE cohort was genotyped and analyzed genome-wide for CNVs [15], and these data were used here to assess the frequency of 15q24 deletions. The AGRE sample comprised 1336 subjects (1051 males, 285 females); 291 were sporadic cases and 494 were from multiplex families (two or more affected children per family).

### Genotyping on oligonucleotide arrays

The CVCR sample was genotyped using *Nsp*I 250K arrays (Affymetrix, Santa Clara, CA, USA). Genotyping was carried out at the University of California Los Angeles DNA Microarray Facility, part of the NIH Neuroscience Microarray Consortium, using manufacturer-recommended procedures for probe generation and hybridization. Analysis of these microarray data for CNVs was carried out as described previously [16], using the dChip software package https://sites.google.com/site/dchipsoft/.

Analysis of 15q24 CNVs in the AGRE cohort was performed in a subset of subjects that had been genotyped (Infinium II HumanHap550 BeadChip, Ilumina, Inc., San Diego, CA) and passed strict quality control parameters, as described in detail previously [15].

Cases with apparent deletions were confirmed with array comparative genomic hybridization (aCGH) and qPCR.

### aCGH

Genomic DNA was purified using a commercial kit in accordance with the instruction manual (DNA Clean & Concentrator-5 Kit; Zymo Research, Orange, CA, USA). aCGH was performed on microarrays according to the manufacturer's instructions (Agilent SurePrint G3 Human CGH 1 × 1 M; Agilent Technologies, Santa Clara, CA, USA). In brief, 1.5 μg of experimental and gender-matched reference DNAs (Promega, Madison, WI, USA) were digested with *Alu*I and *Rsa*I restriction endonucleases (Promega) and fluorescently labeled with cyanine 5-dCTP (Cy-5; experimental) and cyanine 3-dCTP (Cy-3; reference) using a labeling kit (Genomic DNA Labeling Kit; Agilent Technologies). Labeled experimental and reference DNAs were purified, combined, denatured, pre-annealed with Cot-1 DNA (Invitrogen, Carlsbad, CA, USA) and blocking reagent (Agilent Technologies) and hybridized to the microarrays in a rotating oven (20 rpm) at 65°C for 40 hours. After hybridization and recommended washes, the arrays were scanned at 3 μm resolution with a G2505B Agilent Microarray Scanner. Images were processed with Feature Extraction 9.5.1 Software and the data analyzed with DNA Analytics 4.0 software (both from Agilent Technologies). Aberrations were identified using the Aberration Detection Method-1 algorithm with a sensitivity threshold of 6.0 and a data filter that rejected aberrations that did not include at least 10 probes with a log_2 _ratio ± 0.25.

### qPCR

We used the Universal Probe Library (UPL) system (Roche, NJ, USA) to perform genomic qPCR in probands with apparent deletions and their parents, as described previously [16]. Primers were designed with ProbeFinder v2.35 software (Roche, http://www.universalprobelibrary.com).

### Multiplex ligation-dependent probe amplification

The methylation status of the 15q11-q13 Prader-Willi syndrome/Angelman syndrome critical region was assessed by methylation-sensitive multiplex ligation-dependent probe amplification (MLPA) (ME028 PWS/AS MLPA kit; MRC-Holland, Amsterdam, the Netherlands). The kit contains five probes to assess methylation status; four are located in *SNRPN *and one in *NDN*. Electrophoresis of PCR products was performed using an automated sequencer (ABI 3730; Applied Biosystems, Foster City, CA, USA). MLPA data were analyzed using GeneMarker 1.70 software (SoftGenetics, State College, PA, USA).

## Results

### 15q24 microdeletion in a patient from the Costa Rican cohort

**Figure 1:**
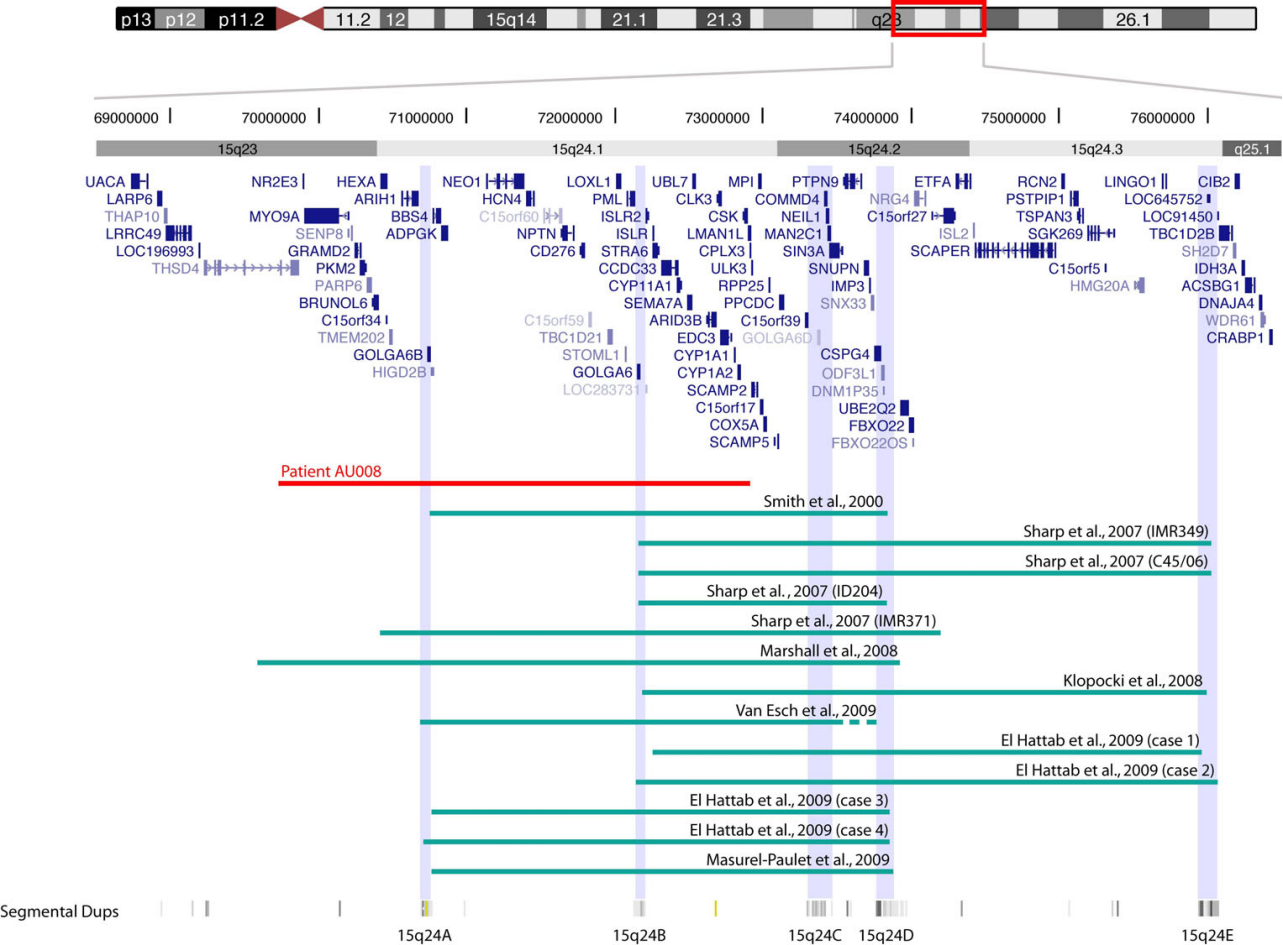
**Map of the 15q23-q24 deletion interval**. Schematic representation of 15q24 microdeletions in patient AU008 and in 13 other patients with overlapping deletions reported previously [2-6,8,9]. The map shows a 8 Mb region in chromosome 15q23-q25.1 (68,500,000-76,500,000, hg18). The vertical bars indicate the five previously reported LCRs, 15q24A-E. Other smaller segmental duplications listed in the UCSC genome browser are shown at the bottom. The minimal critical deletion region identified previously extends 1.75 Mb between LCRs 15q24B (BP1) and 15q24D (BP2). The atypical distal breakpoint in patient AU008 (red) narrows the critical region to 766 Mb, containing only 15 RefSeq genes.

**Figure 2:**
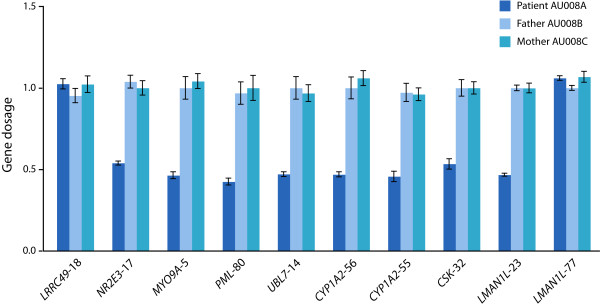
**qPCR gene dosage of the 15q24-15q24.1 region in family AU008**. Genes within the deleted interval and the flanking regions were targeted with qPCR probes in patient AU008 and both parents. The names of the genes are followed by the number of the UPL probe used. Data are means ± SEM. A gene dosage ratio of 1 indicates the presence of two alleles and is considered normal; values < 1 indicate a deletion. The distal breakpoint is located in the *LMANL1 *gene, between intron 1 (probe 23) and exon 14 (probe 77).

**Figure 3:**
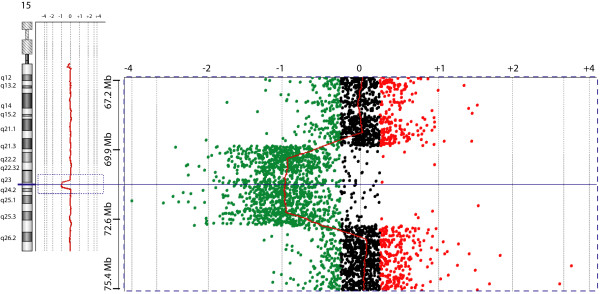
**aCGH showing a 3.1 Mb deletion at 15q23-q24.1 in patient AU008**. Scatterplot (chromosome view) from the 1 × 1 M array using DNA Analytics software (Agilent Technologies). The X axis represents copy number relative to the control and the Y axis represents location along chromosome 15. The area enclosed by the dashed box adjacent to the chromosome 15 ideogram is enlarged on the right. Green circles indicate probes with a log2 ratio ≤ -0.25, red circles denote probes with a log2 ratio ≥ 0.25; black circles are probes that fall in between these two values.

We screened 173 unrelated patients with ASD from Costa Rica using the Affymetrix 250K single nucleotide polymorphism (SNP) microarrays and identified a deletion of the 15q23-q24.1 region in a boy with autism, moderate intellectual disability and mild dysmorphic features (patient AU008). The deletion encompassed at least 2.8 Mb (hg18, chr15:69,897,977-72,697,052), partially overlapping the minimal interval of the recurrent 15q24 microdeletion (Figure 1). Analysis of parental DNA with the 250K SNP arrays revealed that the deletion had occurred *de novo *in the paternal chromosome (data not shown). Real-time qPCR experiments confirmed the deletion in the patient and showed that the distal breakpoint was located in the *LMANL1 *gene, between intron 1 (deleted) and exon 14 (normal) (Figure 2). High-resolution aCGH was performed to characterize further the extent of the deletion, revealing a 3.06 Mb loss (69,837,976-72,896,937) (Figure 3). Neither breakpoint coincides with the recently characterized LCRs in the region [2,3] or with smaller segmental duplications reported in the UCSC genome browser http://www.genome.ucsc.edu/ (Figure 1). The distal breakpoint lies between LCR 15q24B and 15q24C. Therefore, the deletion in this patient would narrow the critical region for the novel 15q24 microdeletion syndrome from 1.75 Mb to 766 kb.

### Clinical description of the patient with an atypical 15q24 deletion

This male child (patient AU008) was 5 years and 1 month old at the time of evaluation. Table 1 provides a summary of the clinical features in the patient compared with 13 other 15q23-q24 deletion carriers. The patient was born at term (39 weeks) to a 21-year-old G1P1 mother and a 28-year-old father. The psychiatric history of the mother's family was notable for the fact that the mother's paternal grandmother, maternal grandfather and a maternal aunt had been diagnosed with schizophrenia, and idiopathic intellectual disability had been reported in a maternal cousin. During pregnancy, polyhydramnios was detected and the mother was hospitalized for several days in the sixth month for pre-eclampsia. Labor was induced with oxytocin; it lasted 6 hours and the delivery was vaginal. At birth, the child weighed 2.46 kg (fifth percentile), was 48 cm long (25th percentile) and had a head circumference of 32 cm (fifth percentile). His Apgar scores were 9 at 1 minute and at 5 minutes after birth. He was described as an irritable baby during the perinatal period.

**Table 1:** Clinical features in Patient AU008 compared to 13 individuals with 15q24 deletions reported in the literature

	Present case	13 previously reported cases	Toatal (%)
**Deletion 15q24**			
Length, MB	3.06	1.7 to 4.3	
Inheritance	*De novo*	11 *de novo *(2 unknown)	
Parental origin	Paternal	2 paternal, 3 maternal (8 unknown)	
**Gender **	M	11 M, 2 F	
**Behavioral abnormalities **			
Developmental delay/ID	Moderate ID	13/13	**14/14 (100%)**
Impaired speech development	Language regression; 2 words at 5 y	7/9	8/10 (80%)
ASD	Autism	2 ASD, 1 autistic features/13	3/14 (21%)
Developmental regression	+	1/12	2/13 (15%)
Happy facial expression	Constant smiling	3/12	4/13 (31%)
Hyperactivity	+	2/12	3/13 (23%)
Aggressiveness	+	2/12	3/13 (23%)
Sleep disturbances	+	1/12	2/13 (15%)
**Growth**			
Low birth weight	+	4/12	5/13 (38%)
Pesistent growth retardation	-	5/12	5/13 (38%)
Obesity	-	3/12	3/13 (23%)
Head circumference <3rd percentile	-	3/12	3/13 (23%)
**Facial dysmorphism **	+	13/13	**14/14 (100%)**
High anterior hair line	+	8/12	9/13 (69%)
Long narrow face	+	4/12	5/13 (38%)
Hypertelorism	+	7/12	8/13 (62%)
Broad medial eyebrows	+	6/12	7/13 (54%)
Epicanthus	+	6/12	7/13 (54%)
Full lower lip	+	5/12	6/13 (46%)
Widely spaced teeth	+	1/12	2/13 (15%)
Ear abnormalities	Protuberant ears	8/12	9/13 (69%)
Facial asymmetry	-	4/12	4/13 (31%)
Down-slanting palpebral fissures	-	7/12	7/13 (54%)
Deep set eyes	-	2/12	2/13 (15%)
Broad nasal base	-	4/12	4/13 (31%)
Flaring alae nasi	-	3/12	3/13 (23%)
Hypoplastic alae nasi	-	2/12	2/13 (15%)
Depressed nasal bridge	-	2/12	2/13 (15%)
Long and/or smooth philtrum	-	8/12	8/13 (62%)
Small mouth	-	3/12	3/13 (23%)
High arched palate	-	2/12	2/13 (15%)
**Eye abnormalities **	+	7/12	8/13 (62%)
Nystagmus	+	1/12	2/13 (15%)
Strabismus	-	6/12	6/13 (46%)
Microphtalmia	-	1/12	1/13 (8%)
Anisocoria	-	1/12	1/13 (8%)
**Digital abnormalities **	+	10/12	11/13 (85%)
Single palmar crease	Right single palmar crease	2/12	3/13 (23%)
Syndactyly	Left 2-3 syndactyly of toes	1/12	2/13 (15%)
Clinodactyly	-	3/12	3/13 (23%)
Long slender fingers	-	2/12	2/13 (15%)
Brachydactyly	-	2/12	2/13 (15%)
Abnormal thumbs	-	2/12	2/13 (15%)
Small hands	-	2/12	2/13 (15%)
Proximally implanted thumbs	-	2/12	2/13 (15%)
**Neurological abnormalities**			
CNS abnormality by MRI/CT	Minimal cortical atrophy on CT	4/7	5/8 (63%)
Hypotonia	Not reported but probable	8/12	9/13 (69%)
Seizures	-	1/12	1/13 (8%)
**Genital abnormalities **	-	8/10 M	8/11 (73%)
Hypospadias	-	4/10 M	4/11 (36%)
Micropenis	-	4/10 M	4/11 (36%)
Cryptorchidism	-	2/10 M	2/11 (18%)
**Musculoskeletal abnormalities**			
Scoliosis	Mild scoliosis	4/13	5/14 (36%)
Joint laxity	+	7/12	8/13 (62%)
Chest abnormalities	-	3/12	3/13 (23%)
Clubfeet	-		
**Other**			
Polyhydramminos	+	0/12	1/13 (8%)
Recurrent infections	Recurrent respiratory infections	6/12	7/13 (54%)
Unusual voice	-	4/12	4/13 (31%)
Diaphragmatic hernia	-	3/13	3/14 (21%)
Inguinal hernia	-	3/12	3/13 (15%)
Growth hormone difficency	-	2/12	2/13 (15%)
Bowel atresia	-	2/12	2/13 (15%)
Hypogonadism	-	2/12	2/13 (15%)
Hearing loss	-	2/12	2/13 (15%)
Café-au-lait spots	-	2/12	2/13 (15%)

CNS, central nervous system; CT, computed tomography; MRI, magnetic resonance imaging.

The child was able to support his head at 15 days, displayed a social smile at 20 days and sat by himself at 6 months. He was able to stand alone at 7 months, but did not walk alone until 30 months, and was unable to hop on one foot. Notably, the parents stated that he did not crawl because he could not tolerate contact with the floor, and that from 1 month of age onwards, he did not want to be touched and would become stiff and throw his head back if someone lifted him in their arms. He acquired sphincter control at 36 months. He spoke his first words at 12 months but never developed phrase speech, eye contact or pointing. The patient had a maximum vocabulary of 10-20 words but stopped using words at the age of 4.5 years. At the time of evaluation, he used only two words.

His parents stated that he had a difficult temper, threw frequent tantrums, and was very restless and hyperactive. He also had difficulty falling and staying asleep. The patient could not modulate his behavior in emotional situations and displayed aggressive behaviors towards himself and/or others when happy or sad. He laughed or cried out of context, and smiled constantly no matter what his mood. He did not respond when called, did not make eye contact and did not follow instructions. He also displayed a multitude of severe restricted and repetitive behaviors. For example, he had difficulty adjusting to change of any kind in his routine or environment. He displayed stereotypies including clapping and hand flapping, and self-stimulating behaviors including spinning, hitting objects or looking at his hands for hours. He put objects in his mouth constantly. He was interested in parts of objects such as the wheels of cars and made frequent meaningless noises.

Evaluation for autism was carried out at 5.1 years of age. The parents were interviewed using the ADI-R and the child was tested with the ADOS module 1 for non-verbal subjects. Both tests showed that the patient fulfilled the criteria for autistic disorder. The results from the ADI-R gave a score of 29 in the reciprocal social interaction area (cut-off is 10), a score of 12 in the communication area (cut-off for non verbal subjects 7) and a score of 8 in the restricted, repetitive behavior area (cut-off 3), with an onset before 36 months (score 3, cut-off 1). On the ADOS, the patient scored 7 points in the communication area (autism cut-off 4) and 10 points in the reciprocal social interaction area (autism cut-off 7), for a total score of 17 points (autism cut-off 12). The Bayley Scales of Infant Development showed a performance IQ < 50. In the Vineland Adaptive Behavior Scales, the child obtained a score of 46 for communication, 52 for daily living skills, 48 years for socialization and 47 for motor skills; the adaptive behavior composite score was 45.

The medical history for this patient is remarkable for chronic allergic rhinitis, asthma and frequent respiratory infections. He also required hospitalization for treatment of several severe dental cavities at the age of 4 years. An ophthalmic examination revealed congenital nystagmus. Brainstem auditory evoked potentials were normal. Computed tomography of the brain revealed minimal cortical atrophy but no other abnormalities. The child was evaluated for sinus bradycardia at 3 and 5 years, but the results of Holter monitoring were normal and there was no evidence of arrhythmias. A spinal X-ray revealed slight lumbar scoliosis but was otherwise normal. Although the patient did not present with typical Angelman features, the inappropriate laughter led us to test him for this syndrome. A 15q11-q13 deletion was ruled out by the CNV analysis. The methylation status of the Prader-Willi syndrome/Angelman syndrome critical region in 15q11-q13 was assessed using methylation-sensitive MLPA and no abnormalities were observed, thus ruling out a uniparental disomy or imprinting defect. Tests were negative for the *FRAXA *mutation and there was a normal G-banded karyotype at 550-band resolution. Routine blood chemistries, full blood count, and urine analysis were also normal.

**Figure 4:**
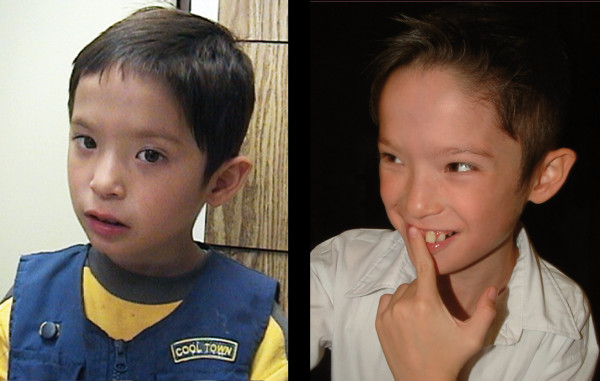
**Photographs of patient AU008 with a 15q24 microdeletion**. Patient AU008 at (left) 5.1 years and (right) 8.10 years of age. Note the high anterior hairline, long narrow face, broad medial eyebrows, epicanthal folds, hypertelorism, full lower lip, widely spaced teeth and protuberant ears. Specific consent from the parents was obtained to publish these photographs.

On physical examination at 5.1 years, the patient weighed 17.5 kg (30th percentile), measured 106 cm in height (21th percentile) and had a head circumference of 51 cm. He presented with a high anterior hairline, long narrow face, broad medial eyebrows, epicanthal folds, hypertelorism, a full lower lip, widely spaced teeth, and protuberant, posteriorly rotated ears (Figure 4). The examination was otherwise notable for the presence of a right single palmar crease and partial syndactyly of the second and third toes of the left foot. He also exhibited joint laxity. All other aspects of the examination, including evaluation of the heart, abdomen, genitalia and skin, were normal.

### 15q24 microdeletions in the AGRE cohort

Our study in the Costa Rican cohort suggested an estimated frequency of 15q24 deletions in ASD of 1 in 173, but this was a modest sample. For this reason, follow-up studies were carried out in an independent cohort. We made use of recent CNV data from 1,336 AGRE cases (from 785 distinct families) that passed strict quality metrics for CNV calling [15]. We did not observe any large (>100 kb) deletions in this cohort across the minimal 15q24 interval as defined by Sharp *et al. *[2]. We observed and confirmed only a 108 kb deletion (73,911,628-74,020,037) at the distal (telomeric) side of the critical region, encompassing the *UBE2Q2 *and *FBX022 *genes, in two affected brothers, AU058-003 and AU058-004 (see Additional file 1, Figure S1A). The deletion was inherited from the healthy father (AU058-002) and was absent in the mother (AU058-001). The deletion was confirmed by qPCR (Additional file 1, Figure S1B). The segregation pattern of this microdeletion and the fact that it lies outside the critical region indicate that it is unlikely to be pathogenic.

## Discussion

### 15q24 microdeletion syndrome

The phenotype of the 15q24 deletion is heterogeneous, but all 14 cases described to date (including the present case) have intellectual disability ranging from mild to severe, and characteristic facial features (Table 1; Additional File 2), suggesting that the 15q24 deletion phenotype may be clinically recognizable [2]. Facial characteristics include high anterior hairline, broad medial eyebrows, hypertelorism, downslanted palpebral fissures, epicanthus, long and smooth philtrum, full lower lip and abnormal ears. Other common features include minor digital anomalies (85%), impaired speech development (80%), genital abnormalities in males (73%), hypotonia (69%), eye abnormalities such as strabismus and nystagmus (62%), joint laxity (62%) and recurrent infections (54%). Less commonly reported features include low birth weight, growth hormone deficiency, diaphragmatic and inguinal hernias, scoliosis and other musculoskeletal abnormalities, bowel atresia, hearing loss and major central nervous system abnormalities (dysplastic corpus callosum with a transected pituitary stalk, myelomeningocele with hydrocephalus and multiple cysts of the corpus callosum) [2,3,6].

In this study, we detected a 3.06 Mb *de novo *deletion of 15q24 of paternal origin in a boy with overlapping clinical features with the previously reported cases of 15q24 microdeletion, including low birth weight, developmental delay, distinct facial features, digital, eye and ear abnormalities, severely impaired language, joint laxity, scoliosis and recurrent respiratory infections. The association with nystagmus is in agreement with an earlier report [2]. Although hypotonia had never been described in the present case, he had delayed motor development, did not walk independently until 30 months and received physical therapy for 4 years, suggesting that he might have been mildly hypotonic. Our patient had no genital abnormalities, whereas hypospadias, micropenis or crytoptorchism have been reported in 8 of 10 previously reported males [2-6].

In addition, patient AU008 exhibited classic features of autism, along with constant smiling and inappropriate laughter, reminiscent of Angelman syndrome. Of the four male cases with a microdeletion of 15q24 initially reported by Sharp *et al.*, three were noted to have happy facial expressions, whereas the fourth subject was described as having 'autistiform' traits [2]. Review of the literature identified two other patients with ASD carrying 15q24 deletions, thus adding this microdeletion syndrome to the increasing list of rare genomic disorders involved in the etiology of autism. Smith *et al. *described a girl with autism carrying an interstitial deletion of chromosome 15q originally mapped by FISH to 15q22-q23 [8]. However, a recent reassessment of this patient using Affymetrix SNP 6.0 microarrays identified a 3.12 Mb deletion (70.740-73.860 Mb) between LCR15q24A and 15q24D (Moyra Smith, personal communication). The girl's phenotype was very similar to that observed in our patient; both exhibited classic autism, intellectual disability, delayed motor development and mild dysmorphic facial features. In addition, both had language regression and were nonverbal at the time of evaluation, they showed prominent mouthing behavior and they had frequent infections. The second patient with ASD carrying a 15q24 microdeletion was identified by Marshall *et al. *in a whole-genome CNV analysis, and limited clinical information was provided [9]. This child appeared to have a more severe phenotype, with severe dysmorphism, severe scoliosis and diaphragmatic hernia (see Additional File 2). The real frequency of ASD among 15q24 deletion carriers is unknown, as the majority of patients reported to date were not formally evaluated for autism. The fact that only some patients with 15q24 deletion have ASD is similar to observations in many other microdeletion and microduplication syndromes, and is part of the variable clinical presentation that can be observed in these genomic disorders. Examples of where ASD can be part of the phenotype at varying rates include 15q11-q13 duplication, 22q13 deletion, Angelman, DiGeorge, Potocki-Lupski and Williams syndromes, to name but a few [17,18]. Other behavioral traits reported in 15q24 deletions include hyperactivity/attention deficit-hyperactivity disorder in two patients, aggressiveness in two and sleep difficulties in one, suggesting that this deletion may confer susceptibility to other neuropsychiatric disorders.

Although it is difficult to perform detailed genotype-phenotype correlations given the limited number of patients described to date, some observations are beginning to emerge. Notably, of the 14 cases with deletions of chromosome 15q24, 12 are males [2-6,8,9]. This distortion in the sex ratio raises the possibility that the penetrance may be influenced by the sex of the individual. Although the phenotype in the two females [3,8] does not appear to differ significantly from that observed in males (see Additional File 2), the apparently biased sex ratio is intriguing, and needs to be confirmed as additional patients are reported. Diaphragmatic hernia has been described in three patients with deletions involving the LCR15q24A-15q24B interval [2,5,9], but was not observed in patient AU008 or in four other patients with deletions affecting the same interval [3,6,8], suggesting incomplete penetrance.

### Frequency of 15q24 microdeletions in ASD

In our study of the Costa Rican sample, we observed one case of 15q24 microdeletion syndrome in 173 unrelated cases ascertained for ASDs. This corresponds to a frequency of 0.54%. This compares to a frequency of 0.23% (1/427) in the study of Marshall *et al. *and a frequency of 0% (0/785 unrelated cases) in the AGRE families. Combining these studies results in an overall frequency of 15q24 microdeletions in patients ascertained for ASD of 0.14% (2/1385). It should be noted that both the AGRE cohort [15] and the Canadian cohort [9] included large proportions of multiplex families (63% and 44%, respectively). Given that in all cases where parental samples were available, 15q24 microdeletions have been shown to be *de novo*, it is likely that rates are higher in simplex families, thus, the rate of 0.14% should be considered as a lower bound. The rates observed in samples ascertained for ASD were in the same order of 15q24 microdeletions observed in patients ascertained for unexplained intellectual disability and congenital anomalies (~0.33%; 4/1200) [2].

### Parental origin of 15q24 deletions

In the three cases with ASD reported to date, the deletion arose in the paternal chromosome (the present case; the girl described by Smith *et al. *[8] (Moyra Smith, personal communication) and the case reported by Marshall *et al. *[9]). By contrast, three of the subjects described by Sharp *et al. *[2] (two with happy facial expression and one with autistic traits), carried deletions of maternal origin. Parental origin was not determined in the remaining cases. It is thus unclear at present the relevance, if any, of the gender of the parent of origin to the resultant behavioral phenotype, and more information is needed. However, it should be noted that no imprinted genes have been described in the deleted 15q23-q24 interval http://www.geneimprint.com/.

### Atypical 15q24 microdeletion breakpoints

Breakpoints of the 15q24 microdeletions described to date are typically defined by segmental duplications, which predispose to recurrent chromosomal rearrangements via non-allelic homologous recombination [2-6]. However, both breakpoints in our patient lie outside these segmental duplications. Similarly, Marshall *et al. *[9] reported a subject with ASD carrying a deletion of 15q23-q24.2, with the proximal breakpoint lying outside the segmental duplications and within the *THSD4 *gene, as in the patient described here (Figure 1), and extending distally to LCR15q24D. However, the breakpoints in *THSD4 *appear to be different, at 69.60 Mb (intron 6) in the subject described by Marshall *et al. *[9] and at 69.84 Mb (intron 14) in patient AU0008. Finally, one of the patients described by Sharp *et al. *(IMR371) had an atypical 15q24 deletion (Figure 1), and precise mapping of both breakpoints and sequencing of the junction fragment revealed two unique breakpoints that were not located within repetitive sequences and had no apparent pairwise homology [2]. The presence of atypical deletions provides an important opportunity to better understand the genes in the minimal region and their relationship to phenotype.

### Genes in the minimal deletion interval

Despite the varying sizes of the 15q24 deletions reported, no correlation has been observed between the extent of the deletion and clinical severity [2-6,8,9]. In fact, the phenotypic similarities between all patients suggest that haploinsufficiency of one or several genes within the minimal deletion interval are responsible for the syndrome. The molecular characterization of the atypical deletion in patient AU008 provides a considerably reduced minimal deletion interval, from 1.75 Mb to 766 kb, implicating a reduced number of genes (15 versus 38 Refseq genes). Of these genes, two have been implicated in autosomal recessive disorders. Homozygous mutations in *STRA6 *result in a multiple malformation syndrome typically associated with anophthalmia or microphthalmia [19], the latter also observed in one patient with 15q24 deletion syndrome [2]. *CYP11A1 *codes for the mitochondrial cholesterol side-chain cleavage enzyme (cytochrome P450scc), catalyzing the first step of steroid biosynthesis leading to production of glucocorticoids, mineralocorticoids and sex hormones. Homozygous mutations in this gene can lead to congenital adrenal insufficiency with disordered sexual differentiation [20].

In addition to *CYP11A1*, two other genes involved in metabolism, *CYP1A1 *and *CYP1A2 *[21], are found in the minimal interval. These genes are involved in the metabolism of endogenous and xenobiotic compounds, including caffeine, theophylline, acetominophen, naproxen and many psychiatric drugs (see the updated list at the Indiana University Drug Interaction table http://medicine.iupui.edu/clinpharm/ddis/table.asp). Careful assessment of drug dosing and sensitivity is therefore warranted in 15q24 deletion carriers. As these enzymes are also important in the metabolism of xenobiotics including environmental toxins, both increased and decreased sensitivity to such toxins might be expected (see [21] for review).

Focusing on neural expressed genes, review of the Allen Brain Atlas http://www.brain-map.org expression data on these genes for four brain regions (cerebellum, CB; neocortex, CTX; hippocampal formation, HPF; and amygdala, AMY) shows very high and ubiquitous staining for UBL7 and region-specific, high levels of expression of SEMA7A (CB), ARID3B (CTX, HPF, AMY) and CLK3 (CTX, HPF) (see Additional File 3). Of these, *SEMA7A *(semaphorin 7A), is crucial for proper axon tract formation during embryonic development [22,23] and T-cell-mediated immune function [24]. SEMA7A binds to plexin-C1 and to integrin-β1 [25,26]. There is evidence from knockout mice that the effects of SEMA7A in axon outgrowth are mediated by integrin-β1 and mitogen-activated protein kinase signaling pathways, possibly thereby regulating the actin cytoskeleton [22]. Interestingly, another member of the semaphorin family, *SEMA5A*, was recently implicated in ASDs through gene expression studies [27] and genome-wide association studies [28].

Ingenuity pathway analysis confirmed significant association of genes in the minimal interval with several metabolic processes (drug metabolism, small molecule biochemistry, protein synthesis, lipid metabolism and vitamin and mineral metabolism; maximum *P *values 5.7 × 10-^07 ^to 1.2 × 10-^05^) driven entirely by the three CYP-family genes. One interesting finding is that PRDM5, which is a zinc finger protein, binds to seven genes in the interval (*GOLGA6*, *ISLR2*, *ISLR*, *CCDC33*, *UBL7*, *ARID3B *and *CLK3*) and regulates their expression [29], indicating coordinated regulation of expression, in turn suggesting an as yet unidentified common functionality. Disruption of multiple genes in a common pathway is more likely to lead to observable phenotypes.

## Conclusions

We estimate that 15q24 microdeletions are etiologically significant in ≥~0.1% of patients with ASDs. The atypical breakpoints observed in our patient narrow the minimal region of overlap and identify genes for sequencing in additional patients. The 15q24 microdeletion appears to be more common in males than females (12 versus 2), and the three patients with ASD reported to date all have paternally derived deletions; these two preliminary observations deserve further investigation.

## Competing interests

The authors declare that they have no competing interests.

## Authors' contributions

LAM, PJG and ERM were responsible for recruitment of patients from Costa Rica. PJG and MF provided the clinical work up of patient AU008A and worked with ERM and CB to prepare the description in the manuscript. LAM was responsible for the survey of CNVs in the Costa Rican families, and LAM, AN, MP, TB, LE and CB were responsible for detailed analysis of the CNV in patient AU008A. JG and HH provided extensive data on CNVs in the 15q24 region in the AGRE cohort. MP, TB, LE and CB were responsible for validating the CNVs in the AGRE families. LAM, CB and JDB were responsible for the design and analysis of experiments, and for the preparation and editing of the manuscript, tables and figures.

## Supplementary Material

Additional file 1 **Figure S1. Deletion of the *UBE2Q2 *and *FBX022 *genes in 15q24.2 in family au058**. **(a) **Schematic representation of the 15q24 microdeletion identified with the Illumina 500 k SNP microarray in AGRE Family AU058. The map shows a 860 kb region in chromosome 15q24.2 (hg18 chr15: 73,540,000-74,400,000). The 108 kb deletion was present in two affected brothers (AU058-003 and AU058-004) and in their healthy father (AU058-002). The vertical bar indicates the LCR cluster 15q24D (BP2). Other smaller segmental duplications listed in UCSC are shown at the bottom. **(b) **The deletion was confirmed by qPCR using a probe in the *UBE2Q2 *gene in the two patients and their father; the mother (AU058-001) had normal gene dosage. Data represent mean ± SEM. Click here for file

Additional file 2 **Supplementary Table 1.** Detailed clinical features in the present case and in 13 individuals with 15q24 deletions reported in the literature. Click here for file

Additional file 3 **Supplementary Table 2.** Genes within the 15q24 microdeletion critical interval. Refseq genes in the minimal deletion interval defined by the atypical deletion in patient AU008. Click here for file
