# Supplementary figures and images for: A large-scale survey of the novel 15q24 microdeletion syndrome in autism spectrum disorders identifies an atypical deletion that narrows the critical region
Source: Mol Autism. 2010 Mar 19;1:5. doi: 10.1186/2040-2392-1-5 (PMC2907565; doi:10.1186/2040-2392-1-5)

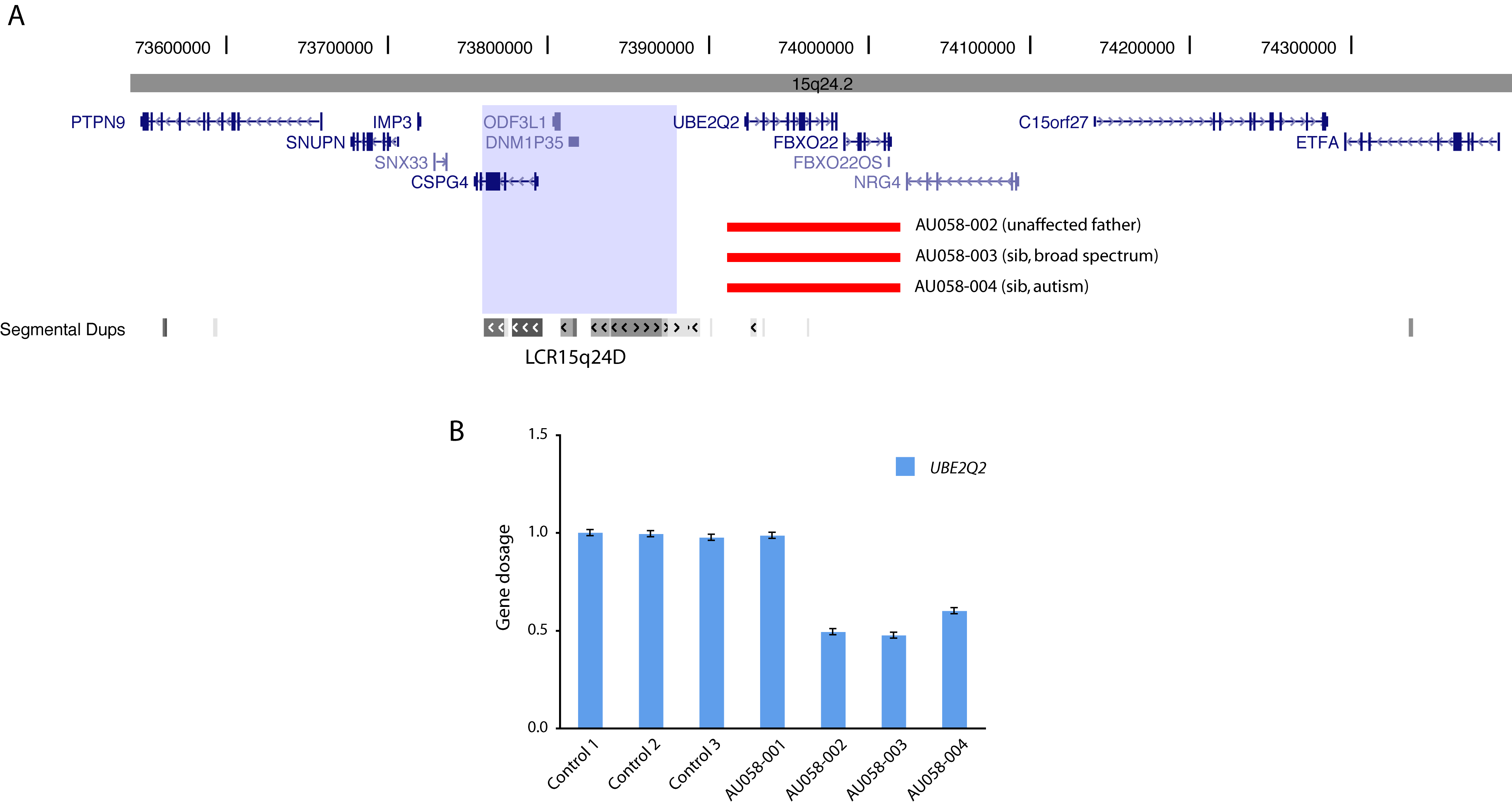

Supplement: Additional file 1 — Figure S1. Deletion of the UBE2Q2 and FBX022 genes in 15q24.2 in family au058. (a) Schematic representation of the 15q24 microdeletion identified with the Illumina 500 k SNP microarray in AGRE Family AU058. The map shows a 860 kb region in chromosome 15q24.2 (hg18 chr15: 73,540,000-74,400,000). The 108 kb deletion was present in two affected brothers (AU058-003 and AU058-004) and in their healthy father (AU058-002). The vertical bar indicates the LCR cluster 15q24D (BP2). Other smaller segmental duplications listed in UCSC are shown at the bottom. (b) The deletion was confirmed by qPCR using a probe in the UBE2Q2 gene in the two patients and their father; the mother (AU058-001) had normal gene dosage. Data represent mean ± SEM. [file 2040-2392-1-5-S1.PNG]
