# Supplementary material for: A large-scale survey of the novel 15q24 microdeletion syndrome in autism spectrum disorders identifies an atypical deletion that narrows the critical region
Source: Mol Autism. 2010 Mar 19;1:5. doi: 10.1186/2040-2392-1-5 (PMC2907565; doi:10.1186/2040-2392-1-5)
Supplement: Additional file 2 — Supplementary Table 1. Detailed clinical features in the present case and in 13 individuals with 15q24 deletions reported in the literature. [file 2040-2392-1-5-S2.PDF]

Supplementary Table 1. Clinical features of individuals with deletions of 15q24

	Sharp et al. (2007)<br>IMR349	Sharp et al. (2007)<br>C45/06	Sharp et al. (2007)<br>ID204	Sharp et al. (2007)<br>IMR371	Klopocki et al. (2008)	Van Esch et al. (2009)	El-Hattab et al. (2009)<br>Case 1
**Del 15q24**	72.15–76.01 Mb	72.15–76.01 Mb	72.15–73.85 Mb	70.40–74.21 Mb	72.2–75.9 Mb	70.6–73.7 Mb	72.252–75.937 Mb
**Length**	3.86 Mb	3.86 Mb	1.7 Mb	3.72 Mb	3.7 Mb	3.1 Mb	3.686 Mb
**Inheritance**	*De novo*	*De novo*	Unknown	*De novo*	*De novo*	*De novo*	*De novo*
**Parental origin**	Maternal	Maternal	Unknown	Maternal	NA	NA	NA
**Age**	14 y	14 y	33 y	15 y	10 y	33 y	33 m
**Gender**	M	M	M	M	M	M	M
**ASD**	Autistic features	—	—	—	—	—	—
**Language**	Speaks well	Few words	Speaks well	Simple speech	Speech delay	Limited speech<br>Echolalia<br>Articulation difficulties	NA
**Development**	Mild MR	Mild MR	Mild MR	Mild MR	Mild MR	Severe MR	Mild delay
**Happy facial expression**	—	+	+	+	NA	NA	NA
**Other behavior**	ADHD	—	—	Sleep disturbances	NA	Hyperactivity<br>Aggressiveness	NA
**Growth**	Height <P3<br>Low birth weight (<P3)<br>HC <P3	Height <P3<br>Low birth weight (<P5)<br>HC <P3	Height <P1<br>Low birth weight (<P2)<br>HC <P3	Height P25<br>Weight P75<br>HC P50	Height P50<br>Truncal obesity<br>HC P75	Tall (185 cm)<br>Obese (94 kg)<br>HC >P97 P (60.5 cm)	Short stature
**Facial features**	High anterior hair line<br>Long narrow face	High anterior hair line<br>Facial asymmetry	High anterior hair line<br>Facial asymmetry	High anterior hair line<br>Facial asymmetry<br>Long narrow face	High anterior hair line<br>Coarse face<br>Full cheeks	High anterior hair line<br>Long face<br>Small maxilla<br>Large mandible<br>Full cheeks	High anterior hair line<br>Broad forehead
**Eye**	Broad medial eyebrows<br>Hypertelorism<br>Down-slanting palpebral fissures	Broad medial eyebrows<br>Hypertelorism<br>Down-slanting palpebral fissures<br>Strabismus<br>Microphtalmia	Broad medial eyebrows<br>Hypertelorism<br>Down-slanting palpebral fissures<br>Deep set eyes<br>Epicanthus<br>Strabismus	Broad medial eyebrows<br>Hypertelorism<br>Long palpebral fissures<br>Nystagmus	Broad medial eyebrows<br>Down-slanting palpebral fissures<br>Periorbital fullness<br>Deep set eyes<br>Strabismus<br>Astigmatism	Broad medial eyebrows<br>Hypertelorism<br>Down-slanting small palpebral fissures<br>Strabismus	Sparse eye brows<br>Epicanthus
**Nose**	Broad nasal base<br>Flaring alae nasi	Broad nasal base<br>Flaring alae nasi	Small nose<br>Hypoplastic alae nasi	High nasal bridge<br>Broad nasal base<br>Flaring alae nasi	Hypoplastic nostrils	Broad nasal base	Depressed nasal bridge
**Mouth**	Long philtrum<br>Full lower lip<br>High arched palate<br>Crowded teeth	Smooth philtrum<br>Full lower lip	Long smooth philtrum<br>Full lower lip	Long philtrum<br>Full lower lip	Long smooth philtrum	Long smooth philtrum<br>High palate<br>Bifid uvula	Long smooth philtrum<br>Full lower lip
**Ear**	Ear abnormalities<br>Hearing loss<br>Tinnitus	Ear abnormalities	Small everted ears	Normal ears<br>Hearing loss	Normal appearance	Large ears	Ear lobe pit
**Hands/feet**	Long slender fingers<br>Proximally implanted thumbs<br>Sandal gap<br>Deep plantar creases	Proximally implanted thumbs<br>Single palmar crease	Small hands<br>Single palmar crease<br>Brachydactyly<br>Syndactyly 2-3, 3-4 fingers and 2-3 toes	Hypoplastic right thumb<br>Contractures of fingers<br>Pes cavus<br>Camptodactyly of toes	Broad thumbs<br>Brachydactyly<br>Clinodactyly<br>Delayed carpal ossification	Long slender fingers with distal tapering<br>Clubfeet	Small hands
**Neurologic**	Wide basal cisterna on brain MRI	Neonatal hypotonia<br>Seizures<br>Brain MRI: dysplastic corpus callosum, pituitary stalk transection, ectopic neurohypophysis, hypoplastic adenohypophysis	— (no MRI)	— (no MRI)	Hypotonia<br>Normal brain CT	Hypotonia<br>Delayed motor development<br>Normal brain imaging	Hypotonia
**Genital**	Normal	Hypospadias	Hypospadias	Hypospadias	Micropenis<br>Cryptorchidism	Micropenis<br>Cryptorchidism	Normal
**Musculoskeletal**	Scoliosis<br>Joint laxity	Scoliosis	Joint laxity	Joint laxity<br>Narrow chest<br>Valgus deformity at ankles	Joint laxity<br>Lumbar lordosis<br>Genua valga	Scoliosis	—
**Respiratory**	Recurrent ear infections	Low tone voice	Recurrent upper airway infections	Recurrent chest infections<br>Asthma	Nasal speech<br>Hoarse voice	High pitched voice	—
**Other**	Growth hormone deficiency<br>Delayed puberty<br>Bowel atresia<br>Coarse hair of two different colors<br>Hairy elbows	Edema of extremities<br>Growth hormone deficiency<br>Hypogonadotropic hypogonadism<br>Bowel atresia	Feeding difficulties as a child	Diaphragmatic hernia<br>Inguinal hernia	Inguinal and umbilical hernias<br>Skin laxity<br>Splenomegaly<br>Widely spaced inverted nipples<br>Elevated triglycerides and LDL/HDL ratio	Diaphragmatic hernia<br>Inguinal hernias	Acute lymphoblastic leukemia<br>Feeding difficulties<br>Hepatomegaly

Abbreviations: ADHD, attention deficit-hyperactivity disorder; ASD, autism spectrum disorder; CT, computed tomography; F, female; HC, head circumference; ID, intellectual disability; IUGR, intrauterine growth retardation; M, male; MR, mental retardation; MRI, magnetic resonance imaging; NA, not available (not reported); P, percentile; + present; — assessed and absent; ? not assessed

Supplementary Table 1 continued

	El-Hattab et al. (2009) Case 2	El-Hattab et al. (2009) Case 3	El-Hattab et al. (2009) Case 4	Masurel-Paulet et al. (2009)	Smith et al. (2000)*	Marshall et al. (2008) SK0243-003	Present study Patient AU008
**Del 15q24**	72.130–76.080 Mb	70.750–73.856 Mb	70.708–73.856 Mb	70.755-73.856	70.740-73.860 Mb	69.60-73.89 Mb	69.838-72.897 Mb
**Length**	3.950 Mb	3.106 Mb	3.148 Mb	3.101 Mb	3.120 Mb	4.289 Mb	3.06 Mb
**Inheritance**	Unknown	*De novo*	*De novo*	*De novo*	*De novo*	*De novo*	*De novo*
**Parental origin**	NA	NA	NA	NA	Paternal	Paternal	Paternal
**Age**	5 m	14 y	9 y	4 y	7 y	?	5 y
**Gender**	F	M	M	M	F	M	M
**ASD**	—	—	—	—	Autism	ASD	Autism
**Language**	NA	NA	NA	2 words only	Language regression, nonverbal	Severe delay	Language regression, nonverbal (2 words)
**Development**	MR	Delayed	Moderate MR	Moderate to severe MR	Severe global delay	Severe MR	Moderate MR
**Happy facial expression**	NA	NA	NA	—	—		Constant smiling
**Other behavior**	NA	NA	Aggressiveness	?	Chewing and mouthing behaviors		Hyperactive/attention deficits<br>Sleep disturbances<br>Aggressiveness<br>Mouthing behavior
**Growth**	Short stature	Normal growth	Obesity	IUGR<br>Height P50<br>Weight P10<br>HC P10	Normal birth weight<br>Height P10<br>Weight P5<br>HC P50		Low birth weight (P5)<br>Normal growth afterwards
**Facial features**	Frontal bossing<br>Brachycephaly	Normal appearance	Facial asymmetry<br>Round face	High anterior hair line<br>Micrognathia	Long narrow face<br>Pointed chin	Severe dysmorphism (no details)	High anterior hair line<br>Long narrow face
**Eye**	Hypertelorism<br>Epicanthus	Down-slanting palpebral fissures<br>Epicanthus	Normal appearance	Sparse eyebrows<br>Down-slanting palpebral fissures<br>Epicanthus<br>Hypertelorism<br>Strabismus<br>Anisocoria	Epicanthus<br>Strabismus		Broad medial eyebrows<br>Epicanthus<br>Hypertelorism<br>Congenital nystagmus
**Nose**	Broad upturned nasal tip	Normal appearance	Normal appearance	—	Depressed nasal bridge		Normal appearance
**Mouth**	Small mouth	Small mouth	Smooth philtrum	Long philtrum<br>Small mouth<br>Glossoptosis	Thin upper lip<br>Wide dental spacing in the upper jaw<br>Narrow anterior palate		Full lower lip<br>Widely spaced teeth
**Ear**	Normal appearance	Thick, small ears<br>Ear lobe pit	Cup-shaped protruding ears	Anteverted ear lobes	—		Protuberant ears
**Hands/feet**	Overriding second toes<br>Clinodactyly<br>Clubfeet	Long first toes and short second toes	Brachymesophalangy II and V<br>Clinodactyly<br>Pes planus	—	Flat nails		Single palmar crease<br>Syndactyly 2-3 toes
**Neurologic**	Myelomeningocele<br>Hydrocephalus	Hypotonia	Hypotonia	Hypotonia<br>Delayed motor development<br>Brain MRI: mutiple cysts of the corpus callosum	Hypotonia<br>Delayed motor development<br>Regression 29 m<br>Brisk tendon reflexes<br>Normal brain CT		Brain CT: minimal cortical atrophy<br>Delayed motor development
**Genital**	Normal	Hypospadias	Micropenis	Micropenis			Normal
**Musculoskeletal**	—	Joint laxity<br>Pectus carinatum	Joint laxity	Kyphosis	Pectus excavatum<br>Lax fingers	Severe scoliosis	Mild scoliosis<br>Joint laxity
**Respiratory**	—	Recurrent ear infections<br>Velo-pharyngeal insufficiency<br>Soft nasal speech	Recurrent ear infections	—	Frequent infections		Recurrent respiratory infections<br>Asthma<br>Chronic allergic rhinitis
**Other**	Tetralogy of Fallot	Café au lait spots<br>Feeding difficulties	Café au lait spots<br>Acanthosis nigricans	—	Light skin, hair and eyes	Diaphragmatic hernia	Polyhydramnios

* The deletion reported by Smith et al. (2002) was originally mapped by FISH to 15q22-q23, but subsequent analysis using Affymetrix SNP 6.0 microarrays revealed a 15q24 deletion (Moyra Smith, personal communication)
