# Supplementary material for: A large-scale survey of the novel 15q24 microdeletion syndrome in autism spectrum disorders identifies an atypical deletion that narrows the critical region
Source: Mol Autism. 2010 Mar 19;1:5. doi: 10.1186/2040-2392-1-5 (PMC2907565; doi:10.1186/2040-2392-1-5)
Supplement: Additional file 3 — Supplementary Table 2. Genes within the 15q24 microdeletion critical interval. Refseq genes in the minimal deletion interval defined by the atypical deletion in patient AU008. [file 2040-2392-1-5-S3.PDF]

Supplementary Table 2. Genes within the 15q24 microdeletion critical interval

Gene ID	Gene name	Start (hg 18 coordinates)	End	Associated human disease	Brain expression CB	CTX	HPF	AMY
GOLGA6	Golgi autoantigen, golgin subfamily a, 6	72 149 251	72 161 944		ND	ND	ND	ND
ISLR2	Immunoglobulin superfamily containing leucine-rich repeat 2	72 208 768	72 216 196		4.1	3.6	18.1	1.4
ISLR	Immunoglobulin superfamily containing leucine-rich repeat	72 253 140	72 256 265		11.4	3.0	9.4	2.9
STRA6	Stimulated by retinoic acid gene 6 homolog	72 273 784	72 288 424	Homozygous mutations result in a multiple malformation syndrome with anophthalmia	7.1	1.2	5.4	3.4
CCDC33	Coiled-coil domain containing 33	72 315 720	72 415 535		ND	ND	ND	ND
CYP11A1	Cytochrome P450, subfamily XIA, polypeptide 1	72 417 156	72 447 134	Homozygous mutations result in congenital adrenal insufficiency with 46,XY sex reversal	2.7	5.6	3.2	1.8
SEMA7A	Semaphorin 7A	72 488 684	72 513 352		72,0	16.9	8.8	0.5
UBL7	Ubiquitin-like 7	72 525 371	72 540 563		100	100	100	100
ARID3B	AT rich interactive domain 3B	72 620 601	72 677 525		13.3	73.1	42.3	45.8
CLK3	CDC-like kinase 3	72 687 766	72 709 595		25.1	53.2	53.7	17.4
EDC3	Enhancer of mRNA decapping 3	72 709 953	72 775 439		100	32.8	17.1	0
CYP1A1	Cytochrome P450, family 1, subfamily A, polypeptide 1	72 798 936	72 804 930		0.9	0.9	1.3	0.4
CYP1A2	Cytochrome P450, family 1, subfamily A, polypeptide 2	72 828 237	72 835 994		6.7	9.1	6.2	6.0
CSK	c-src tyrosine kinase	72 861 478	72 882 592		15.4	13.8	19.5	15.5
LMAN1L	Lectin, mannose-binding, 1 like	72 892 247	72 905 152		9.8	9.9	8.6	0.8

Mouse brain expression values were derived from the Allen Brain Atlas (http://www.brain-map.org/) and reflect average expression levels over a particular region normalized to 'maximum possible expression' (which is in turn derived from a set of ubiquitously expressed genes). Qualitatively similar results were observed using expression density (defined as the number of expressing cells in a given region normalized to the maximum possible number of expressing cells in the same region, again derived from a set of ubiquitous genes in that region). Abbreviations: CB, cerebellum; CTX, neocortex; HPF, hippocampal formation; AMY, amygdala; ND, not detected.
